# Clinical and laboratory profile and outcomes of hospitalized COVID‐19 patients with type 2 diabetes mellitus in Ghana – A single‐center study

**DOI:** 10.1002/edm2.391

**Published:** 2022-11-25

**Authors:** Yasmine Oladele Hardy, Elena Libhaber, Emmanuel Ofori, Divine Aseye Yao Amenuke, Samuel Amoabeng Kontoh, James Amoah Dankwah, Rita Larsen‐Reindorf, Caleb Otu‐Ansah, Kojo Hutton‐Mensah, Ebenezer Dadson, Sheila Adamu, Kofi Akyerekoh, Fred Stephen Sarfo, Bernard Nkum

**Affiliations:** ^1^ Directorate of Medicine, Komfo Anokye Teaching Hospital Kumasi Ghana; ^2^ School of Medical Sciences Kwame Nkrumah University of Science and Technology Kumasi Ghana; ^3^ School of Clinical Medicine and Health Sciences Research Office, Faculty of Health Sciences University of the Witwatersrand Johannesburg South Africa; ^4^ Department of Pharmacy Practice, College of Health Sciences Kwame Nkrumah University of Science and Technology Kumasi Ghana; ^5^ Ghana Health Service, Ashanti Regional Health Directorate Kumasi Ghana; ^6^ Directorate of Psychiatry, Komfo Anokye Teaching Hospital Kumasi Ghana; ^7^ Directorate of Radiology, Komfo Anokye Teaching Hospital Kumasi Ghana

**Keywords:** clinical profile, COVID‐19, mortality, sub‐Saharan Africa, type 2 diabetes mellitus

## Abstract

**Background:**

In sub‐Saharan Africa and particularly in Ghana, there is scarcity of published literature specifically on the impact of DM on outcomes in COVID‐19 patients. Based on the difference in genetic makeup and demographic patterns in Africans compared to the Western world and with the rising burden of DM and other non‐communicable diseases in Ghana there is a need to define the impact DM has on persons with COVID‐19. This would ensure adequate risk stratification and surveillance for such patients as well as appropriate scale up of therapeutic management if needed.

**Aims:**

This single‐center study describes the clinical and laboratory profile and outcomes of COVID‐19 in‐patients with type 2 diabetes mellitus (DM) in Ghana.

**Materials and Methods:**

Retrospective analysis was undertaken of the medical records of adults with COVID‐19 hospitalized at a facility in Ghana from March to October 2020. Clinical, laboratory and radiological data and outcomes were analysed. Comparisons between COVID‐19 patients with DM and non‐diabetics were done with an independent t‐test or a Mann–Whitney test when normality was not attained. Odds ratios (95% CI) were calculated using univariate logistic regression.

**Results:**

Out of 175 COVID‐19 patients, 64 (36.6%) had DM. Overall mean age was 55.9 ± 18.3 years; DM patients were older compared to non‐diabetics (61.1 ± 12.8 vs. 53.0 ± 20.2 years, *p* = .049). Compared to non‐diabetics, diabetics were more likely to have higher blood glucose at presentation, have hypertension, be on angiotensin 2 receptor blockers [OR, 95% CI 3.3 (1.6–6.7)] and angiotensin converting enzyme inhibitors [OR, 95% CI 3.1 (1.3–7.4)]; and be HIV negative (*p* < .05). Although the values were normal, diabetics had a higher platelet count but decreased lymphocytes, aspartate transaminase and alkaline phosphatase compared to non‐diabetics (*p* < .05). There was no difference in clinical symptoms, severity or mortality between the two groups.

**Discussion:**

The clinical profile of patients studied are similar to prior studies. However the outcome of this study showed that DM was not associated with worse clinical severity and in‐hospital mortality. This could have been due to majority of DM patients in this study having relatively good blood glucose control on admission. Secondly, DM alone may not be a risk factor for mortality. Rather its concurrent existence with multiple co‐morbidities (especially cardiovascular co‐morbidities which may predispose to pro‐inflammatory and pro‐thrombotic states) may be driving the rise in severity and mortality risks reported in other studies. Furthermore, this study was conducted among an African population and Africa has been shown to be generally less severely hit by the COVID‐19 pandemic compared to other regions outside the continent. This has been postulated to be due, among other factors, to inherent protective mechanisms in Africans due to early and repeated exposure to parasitic and other organisms resulting in a robust innate immunity.

**Conclusions:**

This study suggested that DM was not associated with more severe clinical symptoms or worse outcomes among hospitalized COVID‐19 patients. Despite this, it is important that DM patients adhere to their therapy, observe the COVID‐19 containment protocols and are prioritized in the administration of the COVID‐19 vaccines.

**Study highlights:**

In this retrospective, single‐centre study on the clinical and laboratory profile and outcome of hospitalized DM patients with COVID‐19, patients with DM did not have a more severe clinical profile or worse outcomes. They were, however, significantly older, more likely to have higher admission blood glucose, have hypertension, be on angiotensin 2 receptor blockers and angiotensin converting enzyme inhibitors; and be HIV negative compared to the cohort without DM. DM patients should be a priority group for the COVID‐19 vaccines.

## Graphical abstract

This study describes the clinical and laboratory profile and outcomes of hospitalized COVID‐19 patients with type 2 diabetes mellitus (DM) in Ghana. Out of 175 patients (mean age 55.9+/−18.3 years) 64 (36.6%) had DM. Compared to non‐diabetics, DM patients were older (61.1? 12.8 vs. 53.0? 20.2 years, *p* = .049), more likely to have higher blood glucose at presentation, have hypertension, be on angiotensin 2 receptor blockers [OR, 95% CI 3.3 (1.6–6.7)] and angiotensin‐converting enzyme inhibitors [OR, 95% CI 3.1 (1.3‐7.4) 7.4)]; and be HIV negative (*p* < .05). There was no difference in clinical symptoms, severity or mortality between the two groups. Nevertheless,it is important that DM patients adhere to their therapy, observe the COVID‐19 containment protocols and are prioritized in the administration of the COVID‐19 vaccines.

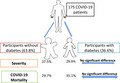

## 1 INTRODUCTION

Since the outbreak of the coronavirus disease 2019 (COVID‐19), caused by severe acute respiratory syndrome coronavirus 2 (SARS‐CoV‐2), cases continued to increase across the globe putting a great burden on health workers and health facilities once it was declared a pandemic.^1^, ^2^ As at 23rd October 2022, 632,672,843 cases had been reported globally with 6,582,222 deaths.^3^

Although type 2 diabetes mellitus (DM) has not been shown to increase the risk of acquiring COVID‐19, it has been identified as one of the risk factors for severe disease and mortality along with increasing age, male sex, cardiovascular disease, hypertension, obesity, malignancies and chronic pulmonary disease among others.^2^, ^4^ Diabetics generally have an increased susceptibility to infections, including respiratory tract infections, with poor prognosis.^5^, ^6^ These result from chronic hyperglycaemia, which leads to a heightened inflammatory state but with a defective T cell response, impaired neutrophil chemotaxis and phagocytic activity and an overall dysfunctional immune system.^5^, ^6^ Furthermore, diabetics tend to have underlying pulmonary impairment.^7^ All these factors may contribute to delayed clearance of SARS‐CoV‐2.

In a cohort of 193 patients with severe COVID‐19 in Wuhan, China, 24.9% had DM and they were in greater need of intensive care unit (ICU) admission, with poorer prognosis compared to their non diabetic counterparts.^8^ Bode et al, in the United States also reported higher morbidity and mortality among hospitalized diabetics with COVID‐19 compared to non‐diabetics.^9^ Some studies in the United Kingdom and North Africa, however, did not report any significant difference in mortality between diabetics and non‐diabetics in their cohorts.^10^, ^11^

There are a number of epidemiological studies and case reports on COVID‐19 patients in sub‐Saharan Africa.^12^, ^13^, ^14^, ^15^, ^16^ However, knowledge of the risk factors for severe disease is still evolving. In sub‐Saharan Africa and particularly in Ghana, there is scarcity of published literature specifically on the impact of DM on outcomes in COVID‐19 patients. Based on the difference in genetic makeup and demographic patterns in Africans compared to the Western world and with the rising burden of DM and other non‐communicable diseases in Ghana,^17^, ^18^ there is a need to define the impact DM has on persons with COVID‐19 so as to ensure adequate risk stratification and surveillance for such patients as well as appropriate scale up of therapeutic management if needed. Taking all these factors into consideration our main objective was to describe the clinical and laboratory profile; and the outcomes of COVID‐19 patients with DM at the Highly Infectious Isolation Unit (HIIU) at the Komfo Anokye Teaching Hospital (KATH). The characteristics of the COVID‐19 cohort with diabetes were also compared with those of COVID‐19 patients without diabetes.

## 2 METHODS

### 2.1 Study design and setting

A retrospective, observational study was carried out at the HIIU at KATH. The medical files of 175 patients, with a definite in‐hospital outcome, admitted at HIIU between the periods of March and October 2020 were consecutively reviewed and the relevant data extracted.

KATH is a teaching hospital, in the Ashanti Region of Ghana, that receives referrals from all over the country. Suspected COVID‐19 cases were admitted to a holding area at the KATH Accident and Emergency center. Those who tested negative for SARS‐CoV‐2 were discharged from the holding area and those with positive results (and severe or critical COVID‐19) were transferred to HIIU for further management. HIIU is an *adhoc*, 23 ‐ bed, isolation unit which was formerly part of the KATH polyclinic but during the pandemic was set up primarily to care for patients with severe confirmed COVID‐19, although occasionally patients with mild and moderate disease were also admitted.

### 2.2 Diagnosis of COVID‐19 and diabetes mellitus

A diagnosis of COVID‐19 was made based on detection of SARS‐CoV‐2 on nasopharyngeal or oropharyngeal swab specimens or sputum using real time‐polymerase chain reaction analysis.^19^ A diagnosis of DM was made on the basis of either an established medical history of DM prior to admission or a persistently elevated random blood sugar ≥ 11.1 mmol/L in the presence of hyperglycaemic symptoms (such as polyuria, nocturia, frequency of urine) or a fasting blood sugar ≥ 7 mmol/L.^20^ COVID‐19 severity was graded according to the COVID‐19 Standard Treatment Guidelines of the Ministry of Health in Ghana.^19^

### 2.3 Data collection

Data from the medical folders of the patients were collected by a team of experienced doctors involved in patient care at HIIU. The data included demographics (age, sex, smoking and alcohol status; and marital status), clinical features, comorbidities, routine baseline laboratory results [complete blood cell count, aspartate aminotransferase (AST), alanine aminotransferase (ALT), alkaline phosphatase (ALP), gamma glutamyl transferase (GGT), urea and creatinine, random blood glucose], serum C‐reactive protein (CRP), chest X‐ray and computed tomography (CT) scan findings; therapy administered and outcomes (hospital transfer, discharge home or death).

CRP was not part of the routine laboratory investigations when the treatment center started operating initially. Therefore, only a small group of patients (10 with DM and 8 non‐DM) had CRP results, and these were performed at an external laboratory at the patients' expense. CT scans (*n* = 31) and chest X‐rays (*n* = 81) were also not done in all patients because some patients (*n* = 20) died within 12–24 h after admission and there were also resource constraints at our facility.

### 2.4 Statistical analysis

Data were entered into an excel sheet. Analysis was performed with Statistica 13.0 and Stata 16.0 packages. Continuous variables were expressed as means and standard deviation (normal distributed variables) and as medians and interquartile range (skewed data). Comparisons between COVID‐19 patients with and without DM were done with an independent t‐test or a Mann–Whitney test when normality was not attained. Categorical data were presented as proportions and percentages and compared using Pearson's Chi‐squared test or Fisher's exact test when appropriate. Bonferoni correction was applied to two‐by‐two comparisons. Univariate logistic regression was used to calculate odds ratio (OR) with the 95% confidence interval (CI). A *p*‐value < .05 was considered significant.

### 2.5 Ethics

Ethical approval was obtained from the KATH institutional review board (IRB) and ethics committee with clearance number KATH‐IRB/AP/067/20. All data collected were anonymized using assigned codes. Informed consent was waived since data were previously collected as part of routine clinical patient care and service delivery.

## 3 RESULTS

### 3.1 Demographics of diabetic and non‐diabetic patients

The mean age of the 175 patients was 55.9 ± 18.3 years, with 54.3% being males. Sixty‐four (36.6%) of the study population was diabetic. Diabetic patients were significantly older than non‐diabetics (61.1 ± 12.8 vs. 53.0 ± 20.2 years, *p* = .049), and there was also a significant difference in marital status (*p* = .034) (Table 1).

**TABLE 1:** Demographic and clinical characteristics of the population

Variables	Total (*N* = 175)	DM (*n* = 64, 36.6%)	Non DM (*n* = 111, 63.4%)	*p* Value
Age (mean ± SD)	55.9 ± 18.3	61.1 ± 12.8	53.0 ± 20.2	.049
Sex (*n*, %)				
Female	80 (45.7)	33 (51.5)	47 (42.3%)	.24
Male	95 (54.3)	31 (48.4%)	64 (57.7%)	
Smoking (*n* = 159)	8 (5.03%)	1 (1.7%)	7 (7.1%)	.26
Alcohol (*n* = 161)	31 (19.3%)	7 (11.7%)	24 (23.8%)	.06
Marital status (*n* = 138)				
Married	90 (65.2%)	34 (65.4%)	56 (65.1%)	.034
Widowed	18 (13.0%)	8 (15.4%)	10 (11.6%)	
Divorced	9 (6.5%)	6 (11.5%)	3 (3.5%)	
Single	21 (15.2%)	4 (7.7%)	17 (19.8%)	
Duration of symptoms before HIIU admission (days) [median (IQR)] (*n* = 173)	7.0 (4.0–14.0)	7.0 (4.0–14.0)	7.0 (4.0–14.0)	.28
Symptoms				
Cough	119 (68%)	44 (68.8%)	75 (67.6%)	.87
Breathlessness	112 (64%)	42 (65.6%)	70 (63.1%)	.73
Fever	89 (50.9%)	32 (0.5%)	57 (51.4%)	.86
Clinical characteristics				
Temperature on admission (deg cent; mean ± SD)	36.9 ± 1.1	36.8 ± 0.9	36.9 ± 1.1	.31
Blood glucose on admission (mmol/L; mean ± SD)	10.1 ± 6.2	15.1 ± 6.8	7.2 ± 3.3	<.001
Systolic blood pressure on admission (mmHg; mean ± SD)	133.1 ± 23.7	136.9 ± 21.1	130.9 ± 24.9	.053
Diastolic blood pressure on admission (mmHg; mean ± SD)	80.8 ± 15.4	80.8 ± 14.4	80.8 ± 16.1	.88
Duration of hypertension (years) (*n* = 42), median (IQR)	9.0 (4.0–11.0)	9.0 (4–11)	8.0 (4.0–13.0)	.94
Duration of DM (years), (*n* = 32) median (IQR)	7.5 (2.5–10.5)	7.5 (2.5–10.5)	–	–
ACE inhibitors	25 (14.3%)	15 (23.4%)	10 (9.0%)	.0086
ARBs	45 (25.7%)	26 (40.6%)	19 (17.1%)	.0006
Severity				
Mild	16 (9.1%)	5 (7.8%)	11 (9.9%)	.50
Moderate	46 (26.3%)	15 (23.4%)	31 (27.9%)	
Severe	53 (30.3%)	24 (37.5%)	29 (26.1%)	
Duration of hospitalization (days) median (IQR)	9.0 (4.0–15.0)	10.0 (4.0–16.0)	9.0 (3.0–14.0)	.28

Abbreviations: ACE, angiotensin converting enzyme; ARBs, angiotensin 2 receptor blockers; deg cent, degree centigrade; DM, type 2 diabetes mellitus; HIIU, Highly Infectious Isolation Unit; IQR, interquartile range; mmol/L, millimoles/litre; SD, standard deviation.

### 3.2 Clinical characteristics

Majority of the study population presented with cough (68%), followed by breathlessness (64%) and fever (50.9%) (Table 1).

Diabetic patients had significantly higher mean levels of blood glucose on admission compared to patients without DM (15.1 ± 6.8 vs. 7.2 ± 3.3 mmol/L, *p* < .0001). Twenty‐two (34.4%) patients with DM had a random blood sugar < 11.1 mmol/L at presentation, [17 (26.6%) had a known medical history of DM and 5 (7.8%) were newly diagnosed]. Only a trend was found for systolic blood pressures between the diabetic and non‐diabetic cohorts (136.9 ± 21.1 vs. 130.9 ± 24.9 mmHg, *p* = .053).

A significant proportion of patients with DM were more likely to be on angiotensin‐converting enzyme (ACE) inhibitors [23.4% vs. 9.0%, OR, 95% CI 3.1 (1.3–7.4), *p* = .0086] and angiotensin 2 receptor blockers (ARBs) [40.6% vs. 17.1%, OR, 95% CI 3.3 (1.6–6.7), *p* = .0006].

No significant difference was found in length of hospital stay between diabetics and non‐diabetics [10.0(4.0–16.0) vs. 9.0 (3.0–14.0, *p* = .28) days, respectively] (Table 1).

### 3.3 Laboratory parameters

The laboratory parameters were not significantly different between the two groups apart from lower lymphocyte count [1.2 vs. 1.5 × 10^3^/μl, *p* = .014], AST [37.7 vs. 50.0 U/L, *p* = .017] and ALP [103.1 vs. 117.9 U/L, *p* = .011] in diabetic patients compared to non‐diabetics and significantly higher platelet counts in the former cohort [239.5 vs. 180.5 × 10^3^/μl, *p* < .0001] (Table 2).

**TABLE 2:** Laboratory parameters of the population

	Normal range	Total	DM	Non DM	*p* Value
Baseline Hb, (*n* = 170) (mean ± SD)	11.5–16.5 g/dl	10.8 ± 2.7	11.1 ± 2.4	10.6 ± 2.9	.37
Baseline WBC (*n* = 170), (median (IQR)	4–10 × 10^3^/μl	8.6 (6.2–12.8)	8.1 (6.8–12.2)	8.9 (6.1–13.4)	.97
Neut count, (*n* = 166), (median (IQR)	1.5–7 × 10^3^/μl	5.9 (4.0–9.6)	6.2 (4.8–10.1)	5.4 (3.7–9.5)	.15
Lymphocyte count, (*n* = 168), (median (IQR)	1–3.7 × 10^3^/μl	1.4 (0.9–1.9)	1.2 (0.9–1.6)	1.5 (1.0–2.1)	.014
AST, (*n* = 148), (median (IQR)	1–40 U/L	48.8 (24.9–78.4)	37.7 (22.7–61.3)	50.0 (29.3–99.6)	.017
ALT, (*n* = 142), (median (IQR)	1–41 U/L	34.5 (19.4–65.7)	38.2 (19.5–63.4)	34.1 (19.1–78.0)	.68
GGT, (*n* = 146), (median (IQR)	6–42 U/L	79.2 (44.9–148.4)	69.7 (44.7–98.1)	87.4 (47.1–163.7)	.06
ALP, (*n* = 146), (median (IQR)	80–305 U/L	114.4 (86.1–153.1)	103.1 (81.1–125.2)	117.9 (91.7–187.3)	011
Urea, (*n* = 158), (median IQR)	2.5–8.3 mmol/L	6.7 (4.0–16.2)	7.2 (4.1–17.6)	6.2 (3.7–16.2)	.34
Creatinine, (*n* = 158), (median (IQR)	44–80 μmol/L	107.0 (75.0–228.0)	116.0 (77.0–217.0)	107.0 (69.0–251.0)	.39
Platelet count, (*n* = 170), (median (IQR)	140–440 × 10^3^/μl	192.5 (128–265)	239.5 (175.5–309.5)	180.5 (107–244)	.0001
Baseline CRP, (*n* = 18), (median IQR)	<5 mg/L	95.3 (16.9–222.5)	233 (95.8–353)	36.7 (3.2–110)	.009
Chest Xray findings (*n* = 81)					
Ground glass opacities		48 (59.3%)	19 (65.5%)	29 (55.8%)	.36
Ground glass opacities and consolidation		16 (19.8%)	6 (20.7%)	10 (19.2%)	
Consolidation only		8 (9.9%)	3 (10.3%)	5 (9.6%)	
Other		9 (9.9%)	1 (3.4%)	8 (15.4%)	
CT scan findings (*n* = 31)					
Mild		2 (6.5%)	1 (10%)	1 (4.8%)	.776
Moderate		5 (16.1%)	2 (20%)	3 (14.3%)	
Severe		24 (77.4%)	7 (70%)	17 (80.9%)	

Abbreviations: ALP, alkaline phosphatase; ALT, alanine transferase; AST, aspartate transferase; g/dl, grams per deciliter; GGT, gamma‐glutamyl transferase; Hb, haemoglobin; IQR, interquartile range; mg/L, milligrams per litre; mmol/L, millimoles per litre; Neut, neutrophils; SD, standard deviation; U/L, Units per litre; WBC, white blood cells; μl, microlitre; μmol/L, micromoles per litre.

Diabetic patients had creatinine levels of 116.0 (77.0–217.0) compared to 107.0 (69.0–251.0) in non‐diabetics, but this did not attain significance.

### 3.4 Comorbidities, therapy administered and outcomes

As shown in Table 3, hypertension was the commonest comorbidity (55.4%) in the whole population followed by diabetes (36.6%), infectious diseases (12.1%) and cardiovascular diseases (10.4%). About a third (33.3%) of the population died; with 29.7% being diabetics and 35.1% non‐diabetics.

**TABLE 3:** Co‐morbidities, therapy administered and outcomes of the population on admission

	Total (*N* = 175)	DM (*n* = 64, 36.6%)	Non DM (*n* = 111, 63.4%)	*p* Value
Co‐morbidities				
Hypertension	97 (55.4%)	55 (85.9%)	42 (37.8%)	<.0001
Infectious diseases	21 (12.1%)	7 (10.1%)	14 (12.8%)	.98
Pulmonary diseases	7 (4.1%)	3 (4.7%)	4 (3.7%)	
Renal diseases	12 (6.94%)	6 (9.4%)	6 (5.5%)	
Neurological diseases	10 (5.8%)	4 (6.3%)	6 (5.5%)	
Gastrointestinal disease	10 (5.8%)	2 (3.1%)	8 (7.3%)	
Genitourinary disease	2 (1.2%)	1 (1.6%)	1 (0.9%)	
Cardiovascular disease	18 (10.4%)	8 (12.5%)	10 (9.2%)	
Others	7 (4.1%)	3 (4.7%)	4 (3.7%)	
Hepatitis B positive (*n* = 99)	9 (9.1%)	3 (8.8%)	6 (9.2%)	1.00
Hepatitis C antibodies (*n* = 83)	2 (2.4%)	0 (0%)	2 (3.9%)	.52
HIV antibodies (*n* = 107)	11 (10.3%)	0 (0%)	11 (15.7%)	.015
Therapy administered				
Hydroxychloroquine	85 (48.6%)	28 (43.8%)	57 (51.4%)	.33
Azithromycin	139 (79.4%)	53 (82.8%)	86 (77.5%)	.40
Zinc/Vit C	95 (54.3%)	32 (40.5%)	65 (67.7%)	.11
Zinc/Vit C/dexamethasone	71 (40.6%)	43 (54.4%)	28 (29.2%)	
Zinc/Vit C/dexamethasone/tocilizumab	5 (2.9%)	4 (5.1%)	1 (1.0%)	
Anti – diabetic agents on admission (*n* = 174)				
Oral antiglycaemics only	27 (15.5%)	27 (42.9%)	0 (0%)	<.0001
Oral antiglycaemics and insulin	23 (13.2%)	23 (36.5%)	0 (0%)	
Insulin only	3 (1.7%)	3 (4.8%)	0 (0%)	
Oxygen needed during admission	112 (64%)	45 (70.3%)	67 (60.4%)	.19
Ventilatory support				
Non invasive (CPAP)	9 (5.1%)	5 (7.8%)	4 (3.6%)	.26
Invasive	5 (2.9%)	3 (4.7%)	2 (1.8%)	
Dialysis	5 (2.9%)	2 (3.1%)	3 (2.7%)	1.00
Outcome				
Hospital transfer	20 (11.4%)	9 (14.1%)	11 (9.9%)	.61
Home	97 (55.4%)	36 (56.3%)	61 (54.9%)	
Death	58 (33.1%)	19 (29.7%)	39 (35.1%)	

*Note*: Others – Skin, Endocrine, Rheumatological and Haematological diseases.

Abbreviations: CPAP, continuous positive airway pressure; Vit C, Vitamin C.

Compared to non‐diabetics, patients with DM were more likely to be hypertensive [85.9% vs. 37.8%, OR, 95% CI 10 (4.5–22), *p* < .0001]. There was also a significant difference with respect to human immunodeficiency virus (HIV) status among diabetics and non‐diabetics (0% vs. 15.7%, *p* = .015) (Table 3).

Regarding anti‐diabetic therapy, 42.9% of diabetic patients were on oral antiglycaemics only and 36.5% were on both oral antiglycaemics and insulin.

We found no significant difference between the diabetic and non‐diabetic cohorts concerning use of a dexamethasone‐based therapy (54.4% vs. 29.2%), tocilizumab‐based therapy (5.1% vs. 1%), oxygen therapy during admission (70.3% vs. 60.4%), severity (37.5% vs. 26.1%) and mortality (29.7% vs. 35.1%) (Table 3).

## 4 DISCUSSION

In this study, the clinical and laboratory profile and the outcomes of patients with DM and SARS‐CoV‐2 infection hospitalized in a single centre in Kumasi, Ghana, were investigated.

We mainly identified that compared to non‐diabetic patients with COVID‐19, patients with DM were older, more likely to have hypertension, a higher blood glucose on admission, be taking ACE inhibitors or ARBs and have a higher platelet count but lower lymphocytes, AST and ALP. In contrast to the recent literature though,^8^, ^9^ DM was not associated with increased in‐hospital mortality.

Over half of our study population were males, and this echoes earlier published literature globally.^21^, ^22^, ^23^ This predilection for men may result from differences in the genetic makeup of men compared to women as well as social lifestyles that are more common in men such as cigarette smoking and alcohol intake; both of which may impair their immune response to infection.^24^

Diabetic patients were significantly older than the non‐diabetic cohort. It has been well‐documented that DM is generally an ageing disease.^25^, ^26^, ^27^ In addition, ageing comes with a decline in respiratory reserve and immune response, which could predispose such patients to acquiring COVID‐19.^28^ Our findings align with that of other researchers who reported that their DM cohort were more advanced in age.^11^, ^29^

Symptoms of cough and breathlessness were observed in high proportion among our COVID‐19 patients with DM, but this did not achieve statistical significance.

Not surprisingly, this study demonstrated that use of ACE inhibitors or ARBs was more commonly seen among diabetics compared to non‐diabetics. Literature shows that an estimated 30% and 60% of all DM patients have albuminuria and hypertension respectively, and if these are not adequately controlled they may result in development of chronic kidney disease and adverse cardiovascular events.^30^, ^31^, ^32^ ACE inhibitors and ARBs confer renoprotective effects by slowing the progression of microalbuminuria and providing good blood pressure control.^32^ Over 80% of our diabetic cohort had hypertension and this may be one reason why they were on these medications. Our study did not assess for the presence of microalbuminuria in our diabetic cohort, but it is possible that some of these patients had developed microalbuminuria earlier and started on ACEi or ARBs by their primary treatment provider. Both ACE inhibitors and ARBs drugs interact with the renin‐angiotensin‐aldosterone system and upregulate ACE 2 expression; the receptor to which SARS‐CoV‐2 binds during cell entry.^33^ Thus, earlier in the pandemic, there was concern that this upregulation would result in increased virulence of SARS‐CoV‐2 with undesirable outcomes but recent studies have contradicted this.^33^, ^34^

Regarding laboratory findings, diabetic patients had significantly higher platelet counts and lower AST and ALP values compared to their non‐diabetic counterparts. Studies have identified that thrombocytopenia,^21^, ^35^ elevated AST^36^, ^37^ and ALP^37^, ^38^ are tied to worsening disease severity and unfavourable outcomes in COVID‐19 patients. Lymphocytopenia is another indicator of disease severity and mortality in COVID‐19.^35^ Though the lymphocytes were significantly lower in diabetics they were however still within the normal range. Lymphocytopenia is due to lymphocyte apoptosis which may result not only from binding of SARS CoV‐2 to the angiotensin converting enzyme (ACE) 2 receptor on lymphocytes but also markedly increased cytokine levels triggered by COVID‐19.^39^, ^40^ Our diabetic population had creatinine levels that were much higher than the normal range. It is possible that this may be due not only to the effects of COVID‐19 but also to pre‐existing, underlying renal impairment which has been reported in 20%–30% of type 2 diabetic patients and which worsens with advancing age.^41^

Although the DM group had an older cohort of patients and age is a risk factor for poor prognosis, there was no significant difference in severity and mortality between the diabetic and non‐diabetic groups. This is in contrast to previous findings that have recorded greater severity and higher mortality rates in their diabetic cohorts compared to non‐diabetics.^8^, ^9^, ^21^, ^42^ Several explanations can be suggested. First, there may be protective genetic mechanisms in our population in contrast to other populations most of whom were from Asia, Europe and the United States.^8^, ^9^, ^21^, ^26^, ^29^, ^43^ Despite its relatively weaker healthcare infrastructure^44^ and contrary to widespread expectations, it is well established that Africa has been relatively less severely hit by COVID‐19 than the afore‐mentioned continents. This finding is postulated to be due in part to innate immunity as well as the protective immunity conferred by repeated exposure to parasitic infections and other coronaviruses in the region.^45^ It is also hypothesised that the bacillus Calmette–Guérin (BCG) vaccine, which is part of the national immunization program for newborns in many countries in sub‐Saharan Africa with a high TB burden, may boost the immune response against SARS‐CoV‐2.^46^, ^47^ In addition, it is likely that DM alone may not be a risk factor for mortality, but rather its concurrent existence with multiple co‐morbidities (especially cardiovascular co‐morbidities which may predispose to pro‐inflammatory and pro‐thrombotic states) may be driving the rise in severity and mortality risks^23^, ^48^, ^49^ Furthermore, about half of the patients with DM had relatively good blood glucose control on admission as evidenced by a random blood glucose of <11.1 mmol/L, and this may also have attenuated their mortality risk. Most of our diabetic cohort were also on metformin, which may have conferred a protective effect due to its documented immunoregulatory effects.^50^ The number of diabetic patients in our study was, however, not large enough for the effect of the individual anti‐diabetic drugs on outcomes to be analysed. Our findings align with that of investigators in the USA.^51^

Among the whole study population, the prevalence of DM was 36.6%. This prevalence observed in our study was much higher than the DM prevalence of 18.7% reported in a Moroccan study and the 14% reported in an earlier study done in Ghana.^11^, ^52^ The higher prevalence we observed could be due to the comparatively larger sample size of our study. The slightly higher prevalence (37.5%), compared to ours, observed by Alkundi et al in the United Kingdom may be as a result of their population being older and therefore more prone to age related conditions like DM.^10^

There are a few limitations of our study that cannot go unmentioned. First, it was a retrospective study and some of the patients admitted at the initial stages of the pandemic had incomplete medical records, especially the laboratory data. Second, the relatively small sample size may have led to failure in detecting an association between some of the variables. In addition, the HbA1C, which would have better captured the level of glucose control, was not done due to resource constraints. Obesity, a comorbidity with prognostic significance, was not included in our analysis because of a lack of data on either weight or height, or both, in a large proportion of the patients.

In conclusion, we found that about a third of our population were diabetic. Compared to non‐diabetic patients, those with DM were older, had higher blood glucose on admission, were more likely to have hypertension, negative HIV serostatus, be taking ACE inhibitors or ARBs and have a higher platelet count but lower lymphocytes, AST, and ALP. However, DM was not associated with in‐hospital mortality. Our study helps fill gaps regarding the outcome of hospitalized patients with DM and COVID‐19. Despite the decreased mortality seen among the diabetic cohort in this study, it is important that there should be ongoing public education about adherence to anti diabetic (and other) therapy as well as the COVID‐19 containment protocols. They should also be prioritized in the administration of the COVID‐19 vaccines. Large, powered studies, prospective in nature and involving multiple treatment sites in Ghana are needed to further elucidate the impact of DM on COVID‐19, especially severity and mortality indicators.

## AUTHOR CONTRIBUTIONS

**Yasmine Oladele Hardy:** Conceptualization (equal); methodology (equal); project administration (equal); supervision (equal); validation (equal); writing – original draft (equal); writing – review and editing (equal). **Elena Libhaber:** Data curation (equal); formal analysis (equal); supervision (equal); validation (equal); visualization (equal); writing – original draft (equal); writing – review and editing (equal). **Emmanuel Ofori:** Methodology (equal); project administration (equal); validation (equal); visualization (equal); writing – original draft (equal); writing – review and editing (equal). **Divine Aseye Yao Amenuke:** Writing – original draft (equal); writing – review and editing (equal). **Samuel Amoabeng Kontoh:** Writing – original draft (equal); writing – review and editing (equal). **James Amoah Dankwah:** Conceptualization (equal); writing – original draft (equal); writing – review and editing (equal). **Rita Larsen‐Reindorf:** Writing – original draft (equal); writing – review and editing (equal). **Caleb Otu‐Ansah:** Writing – original draft (equal); writing – review and editing (equal). **Kojo Hutton‐Mensah:** Writing – original draft (equal); writing – review and editing (equal). **Ebenezer Dadson:** Writing – original draft (equal); writing – review and editing (equal). **Sheila Adamu:** Writing – original draft (equal); writing – review and editing (equal). **Kofi Akyerekoh:** Writing – original draft (equal); writing – review and editing (equal). **Fred Stephen Sarfo:** Writing – original draft (equal); writing – review and editing (equal). **Bernard Nkum:** Writing – original draft (equal); writing – review and editing (equal).

## CONFLICT OF INTEREST

The authors declare that they are no conflicts of interest.

## Data Availability

The data that support the findings of this study are available on request from the corresponding author.
